# Creation of an Open Bedside Tracheostomy Program at a Community Hospital With a Single Surgeon

**DOI:** 10.1002/oto2.27

**Published:** 2023-02-17

**Authors:** Ryan S. Ziltzer, Noah M. Millman, Jorge Serrano, Mark Swanson, Karla O'Dell

**Affiliations:** ^1^ Department of Otolaryngology‐Head and Neck Surgery Keck School of Medicine of University of Southern California California Los Angeles USA; ^2^ Department of Emergency Medicine LAC+USC Medical Center Los Angeles California USA; ^3^ Department of Otolaryngology‐Head and Neck Surgery University of Southern California Los Angeles California USA

**Keywords:** community hospital, cost effectiveness, open bedside tracheostomy, quality improvement

## Abstract

**Objective:**

To assess the adverse event rate and operating cost of open bedside tracheostomy (OBT) at a community hospital. To present a model for creating an OBT program at a community hospital with a single surgeon.

**Study Design:**

Retrospective case series pilot study.

**Setting:**

Academic‐affiliated community hospital.

**Methods:**

Retrospective chart review of surgical OBT and operating room tracheostomy (ORT) at a community hospital from 2016 to 2021. Primary outcomes included operation duration; perioperative, postoperative, and long‐term complications; and crude time‐based estimation of operating cost to the hospital using annual operating cost. Clinical outcomes of OBT were assessed with ORT as a comparison using *t* tests and Fisher's exact tests.

**Results:**

Fifty‐five OBT and 14 ORT were identified. Intensive care unit (ICU) staff training in preparing for and assisting with OBT was successfully implemented by an Otolaryngologist and ICU nursing management. Operation duration was 20.3 minutes for OBT and 25.2 minutes for ORT (*p* = .14). Two percent, 18%, and 10% of OBT had perioperative, postoperative, and long‐term complications, respectively; this was comparable to rates for ORT (*p* = .10). The hospital saved a crude estimate of $1902 in operating costs per tracheostomy when performed in the ICU.

**Conclusion:**

An OBT protocol can be successfully implemented at a single‐surgeon community hospital. We present a model for creating an OBT program at a community hospital with limited staff and resources.

Modern medical advancements have led to the ability to sustain critically ill patients and increased the need for tracheotomy in intensive care unit (ICU) patients.^1^ Up to one‐third of ICU patients require mechanical ventilation, which accounts for a disproportionate amount of ICU costs in addition to increasing the risk of nosocomial infections.^2^, ^3^, ^4^ Tracheostomy is a common procedure warranted in patients requiring prolonged mechanical ventilation.^5^ Indications for tracheostomy include prevention of complications related to lengthy translaryngeal intubation, facilitation of weaning from mechanical ventilation, improving oral hygiene, pulmonary toilet, and patient comfort.^6^

Tracheostomy can be surgically performed in the operating room (OR) or at the bedside in the ICU.^7^ The traditional open tracheostomy was originally described by Jackson in 1909, and the standard surgical maneuvers are used in both open operating room tracheostomies (ORTs) as well as open bedside tracheostomies (OBTs).^8^ The major advantage of an open approach is its utility for patients that may not be a candidate for a percutaneous dilatational tracheostomy approach due to body habitus or nonpalpable anatomy.^9^, ^10^ When comparing ORT and OBT, OBT has been demonstrated to have decreased costs and better resource utilization with comparable safety and efficacy.^6^, ^7^, ^11^, ^12^ One study found that time‐to‐surgery was halved for OBT procedures compared to ORT.^6^ OBT demonstrates comparable or lower complication rates in comparison to ORT, even in a highly comorbid population without strict selection criteria.^5^, ^6^, ^7^, ^13^, ^14^ In 1 study, the incidence of significant complications in the 30 days following OBT had been found to be less than 5%.^15^ In another study of OBT, the incidence of major complications, including hemorrhage and myocardial infarction, was less than 1%.^11^ Other advantages of OBT include minimizing the risk of transporting critically ill patients, avoiding OR costs, and decreasing the consult‐to‐procedure time.^5^, ^7^, ^8^, ^12^

Despite the established safety and advantages of OBT,^5^, ^6^, ^7^, ^8^, ^11^, ^12^, ^13^, ^14^, ^15^ the open bedside approach is underutilized nationally.^7^ In addition to concern for complications, lack of staffing is commonly cited by larger academic hospitals as a reason for not implementing OBT.^7^ However, even in the setting of academic‐affiliated community hospitals, uptake of evidence‐based ICU intervention programs can occur regardless of hospital size and interventionist staffing.^16^ Barriers to ICU program implementation at community hospital affiliates include time and financial resources, while facilitators center around better training attendance and program activity completion as well as an engaged, collaborative nursing and physician champion team.^16^, ^17^ Prior literature found that in hospital settings resistant to performing OBT, the establishment of a rigid standardized protocol alleviated staff concerns.^15^ However, the present literature offers little guidance to Otolaryngologists on exactly how to establish an OBT protocol at a community hospital. This pilot study reports on the complication rate, operating cost, and feasibility of OBT at a single‐surgeon community hospital affiliate and presents a model for creating an OBT program at a community hospital. We present this model to aid the community hospital Otolaryngologist interested in implementing an OBT protocol.

## Methods

### Creation of the OBT Program

We present our experience with creating an OBT program at an academic‐affiliated community hospital where there were no physicians performing percutaneous dilatational tracheostomy and no surgeon performing OBT. In 2013 Verdugo Hills Hospital, a community hospital in Glendale, California, partnered with the University of Southern California (USC) to become the community hospital affiliate of USC Verdugo Hills Hospital. At the time, no physicians were performing percutaneous dilatational tracheostomy and all tracheostomies were performed as ORT by Otolaryngologists from the community and, after the partnership with USC, from a large academic medical center. A single surgeon from this pool of rotating Otolaryngologists covers the hospital at a time and operates independently.

In 2016, the OBT program was created at Verdugo Hills Hospital by a single Otolaryngologist who had experience performing OBT at USC's larger academic hospitals. The program began with the creation of an OBT protocol with the Otolaryngology surgeon, nursing administration in the ICU and OR, and hospital leadership. A bedside tracheostomy competency module was developed with a written protocol, detailed diagrams of procedure steps, and video components.^18^, ^19^ All ICU nursing staff, OR nursing staff and scrub techs, and respiratory therapy (RT) were required to pass this module to participate in bedside tracheostomies. The new staff is required to complete this module as part of the onboarding process at this hospital. After training the program was set with a 6‐month trial period to evaluate safety events for this approach prior to approval with the hospital. The program was initially approved by a single surgeon (K.O.) and then expanded to all surgeons with previous bedside tracheostomy experience per hospital privileging requests. The OBT is performed as follows. The ICU critical care physician, in discussion with the patient family, determines when a patient requires a tracheostomy. The case is scheduled by the surgeon with the OR to ensure OR staffing, in coordination with the ICU nursing. The OR staff includes 1 circulating nurse and 1 OR technician who bring a tracheostomy tray, sterile setup, gowns, gloves, headlights, tracheostomy tubes of various sizes, sutures, Bovie machine, and OR table to the ICU bedside. The OR staff set up the room. The procedure is performed with the Otolaryngology surgeon and the OR technician as first assist in the standard open tracheostomy procedure. The ICU nurse is responsible for performing the preoperative checklist, confirming consent, correct the patient and procedure. Most patients in the ICU requiring tracheostomy are already under sedation and managed by the ICU. Patients currently receiving sedation while on mechanical ventilation are continued on their standard sedation and given additional fentanyl or midazolam as needed. One‐percent lidocaine with epinephrine injection for local anesthesia is also given. RT stands at the head of the ICU bed and is responsible for removing the endotracheal tube when the tracheostomy is being placed and reconnecting the patient to the ventilator. RT also records end‐tidal CO_2_ and tidal volume to confirm correct tube placement. After the procedure, tracheostomy care is performed by ICU nursing staff. Tracheostomy sutures are removed by RT on postoperative day 5 and soft tracheostomy ties are placed. ICU critical care physicians and medicine physicians change patients to uncuffed tracheostomy on a postoperative day 5 for patients who no longer require mechanical ventilation.

### Review of Outcomes of the OBT Program

Medical records of patients who underwent surgical (open) tracheostomies at the single‐surgeon community hospital affiliate USC Verdugo Hills Hospital between 2016 and 2021, after the implantation of the OBT program, were identified and retrospectively reviewed. Both OBT and ORT during the study period were included. Tracheostomies for prolonged mechanical ventilation and respiratory failure were included, tracheostomies performed for other indications (eg, laryngectomy) were excluded. The location of tracheostomy (OBT or ORT) was based on the preference of the consulted physician, and patients were not excluded from OBT based on specific criteria. All operating surgeons were Otolaryngologists from the community and from an affiliated academic medical center.

Patient demographics, Charlson Comorbidity Index (CCI) score, body mass index, and prior tracheostomy were recorded. Primary clinical outcomes included duration of operation, perioperative and postoperative complications, and long‐term complications. Duration of operation (“surgery stop time” minus “surgery start time”) was obtained from the official OR record that is generated by the circulating nurse during every surgical procedure. Perioperative complications included minor bleeding, major bleeding requiring transfusion or surgical exploration, and other complications at the time of surgery. Postoperative complications occurred within 7 days of surgery and included bleeding, infection at the stoma site, pneumothorax, recurrent laryngeal nerve damage, tracheostomy decannulation/dislodgement, mucus plugging requiring bronchoscopy, complications requiring reintubation, and mortality. Long‐term complications occurred after postoperative day 7 and included tracheostomy decannulation, tracheoesophageal fistula, tracheoinnominate fistula, hematoma, tracheal stenosis, delayed closure requiring procedural closure, and mortality.

### Cost Analysis

After discussing approaches to cost analysis with hospital financial leadership, crude time‐based estimations for operating costs to the hospital for OBT and ORT using annual operating expenses were recommended by financial leadership for this pilot study. First, key hospital financial administrative personnel calculated the overall annual operating expense—the cost to the hospital to keep a room operational over 12 months—for 1 ICU room and for 1 OR based on the 2021 fiscal year. They then used this to estimate operative expense rates (cost to the hospital per minute) for the ICU and the OR based on the daily hours of operation (determined to be 24 hours for the ICU and 10 hours for the OR). Duration in operating location (“patient out room time” minus “patient in room time”) was obtained from the official OR record for each operation; for OBT, “patient in room time” on the official OR record is consistent with when all required staff and equipment are present and “patient out room time” is consistent with when anesthesia end time. Operating costs per tracheostomy were then calculated by multiplying the operative expense rates by the average durations in operating location (in minutes) for OBT and ORT.

### Statistical Analysis

A limited preliminary analysis was conducted as part of this pilot study to explore comparisons between the OBT and ORT groups. The *t* test was used to compare mean age, CCI, and duration of operation between OBT and ORT groups. Preliminary analysis used Fisher's exact testing to compare clinical characteristics and complications between OBT and ORT given the test's utility in association testing with small sample sizes.^20^ Significance level was set at 0.05, and 2‐sided *p* values are reported. Analyses were conducted using SAS version 9.4 (SAS Institute).

The study was approved by the USC Institutional Review Board (HS‐21‐00645).

## Results

### Characteristics and Clinical Outcomes

A total of 69 tracheostomies during the study period met the criteria for inclusion in the final sample: 55 OBT and 14 ORT. Most ORT in the study period occurred when the OBT program was just beginning. Table 1 reports the group characteristics for OBT and ORT. Participants in both groups appeared to have similar characteristics (*p* > .05).

**Table 1:** Patient Characteristics

Variable	OBT (n = 55)	ORT (n = 14)	*p* value^a^
Age	71.4 (14.0)	66.4 (20.7)	.29
Gender			.55
Male	29 (53%)	9 (64%)	
Female	26 (47%)	5 (36%)	
BMI			>.99
<18.5 (underweight)	6 (11%)	1 (7%)	
18.5‐24.9 (normal weight)	20 (36%)	6 (43%)	
25‐29.9 (overweight)	17 (31%)	4 (29%)	
30‐39.9 (obese)	8 (15%)	2 (14%)	
40+ (severe obesity)	4 (7%)	1 (7%)	
CCI score	4.9 (2.8)	4.7 (2.2)	.82
Prior tracheostomy	1 (2%)	1 (7%)	.37
Tracheostomy tube size			.36
Size 6 cuffed	38 (69%)	8 (57%)	
Size 8 cuffed	14 (25%)	5 (38%)	
Other	3 (4%)	1 (7%)	

Abbreviations: BMI, body mass index; CCI, Charlson Comorbidity Index; OBT, operating room tracheostomy; ORT, operating room tracheostomy.

^a^ *T* test and Fisher's exact test.

Table 2 reports the primary outcomes compared between OBT and ORT. The average duration of operation were 20.3 minutes for OBT and 25.2 minutes for ORT (*p* = .14). In the perioperative period, 2% of OBT and 0% of ORT had complications. The perioperative complications in OBT were related to an event of minor slow‐oozing bleeding after OBT in an ICU patient with prior thrombocytopenia, which resolved with platelet transfusion. Postoperatively, 18% of OBT and 29% of ORT had complications. Ten percent of OBT and 29% of ORT had no long‐term complications. The most common procedure‐related complication in OBT was mucus plugging requiring bronchoscopy in the postoperative period (5%). Though mortality was the most common complication that occurred during the postoperative and long‐term periods, a careful review of all‐cause mortalities revealed there were no procedure‐related mortalities. Mortalities in the sample occurred in critically ill patients with already poor prognoses who received palliative tracheostomy but died from causes unrelated to tracheostomy. Preliminary analysis suggested that perioperative, postoperative, and long‐term complications did not differ between OBT and ORT groups (*p* > .05).

**Table 2:** Clinical Outcomes

Outcome	OBT (n = 55)	ORT (n = 14)	*p* value^a^^,^ *
Operating duration (minutes)	20.3 (8.5)	25.2 (17.2)	.14
ICU length of stay (d)	24.1 (12.4)	22.5 (15.7)	.69
Intubation to tracheostomy time (d)	13.8 (6.5)	14.1 (12.9)	.90
Perioperative complications, n (%)			
None	54 (98%)	14 (100%)	>.99
Minor bleeding	1 (2%)	0 (0%)	>.99
Major bleeding	0 (0%)	0 (0%)	‐
Other	1 (2%)	0 (0%)	>.99
Postoperative complications, n (%)			
None	45 (82%)	10 (71%)	.46
Bleeding			
Minor bleeding	2 (4%)	1 (7%)	.50
Major bleeding	0 (0%)	0 (0%)	‐
Infection at stoma	0 (0%)	1 (7%)	.20
Pneumothorax	0 (0%)	0 (0%)	‐
Recurrent laryngeal nerve damage	0 (0%)	0 (0%)	‐
Decannulation	1 (2%)	1 (7%)	.37
Mucus plugging requiring bronchoscopy	3 (5%)	0 (0%)	>.99
Reintubation	0 (0%)	1 (7%)	.20
Mortality, any cause (<7 d)	6 (11%)	0 (0%)	.33
Other	0 (0%)	1 (7%)	.20
Long‐term complications, n (%)			
None	44 (90%)	10 (71%)	.10
Hematoma	0 (0%)	0 (0%)	‐
Tracheal stenosis	0 (0%)	0 (0%)	‐
Delayed closure	0 (0%)	0 (0%)	‐
Decannulation	1 (2%)	0 (0%)	>.99
Tracheoesophageal fistula	0 (0%)	0 (0%)	‐
Tracheoinnominate fistula	0 (0%)	0 (0%)	‐
Mortality, any cause (>7 d postop)	4 (8%)	4 (29%)	.06
Other	1 (2%)	2 (14%)	.12
Total mortality, any cause, n (%)	10 (18%)	4 (29%)	.46

Abbreviations: OBT, operating room tracheostomy; ORT, operating room tracheostomy.

^a^ *T* test and Fisher's exact test.

*p < .05.

### Hospital Cost Analysis

The estimated hospital operating cost per minute was $12 for the ICU and $53 for the OR (Table 3). Using the average duration in the operating location, crude cost per tracheostomy was estimated at $451 for OBT and $2353 for ORT (Table 3). The hospital saved an estimated $1902 in operating costs per surgical tracheostomy when performed as OBT.

**Table 3:** Operating Cost Analysis

Measure	OBT (n = 55)	ORT (n = 14)
The average duration in operating location (minutes)	37.6 (13.9)	44.4 (20.1)
Operating cost^a^ per minute	$12	$53
Estimated operating cost per tracheostomy	$451	$2353
Difference	−$1902	

Abbreviations: OBT, open bedside tracheostomy; ORT, operating room tracheostomy.

^a^ Based on the 2021 fiscal year annual operating expenses and daily hours of operation.

## Discussion

This pilot study supports the feasibility of implementing an OBT program at a community hospital affiliate by a single Otolaryngology surgeon. OBT had low rates of procedure‐related complications, similar to that of ORT. Additionally, OBT offered potential savings on operating costs to the community hospital by performing tracheostomy in the ICU instead of the OR. These findings are consistent with the existing literature that establish the safety and cost‐efficiency of OBT.^5^, ^6^, ^7^, ^8^, ^11^, ^12^, ^13^, ^14^, ^15^

A bedside tracheotomy cart consisted of a headlight, electrosurgical unit, tracheotomy tray, surgical instruments, drapes, gowns, and tracheotomy tubes used in the OR.^6^ In the study by Sinha et al, 2 Otolaryngologists, 2 ICU nurses, and 1 anesthetist were used in their OBT procedures.^4^ In another study, all OBTs were performed by thoracic surgeons.^15^ ICU RT are also present and have been rigorously trained and familiar with the nuances of the procedure.^6^ Tang et al described the successful implementation of an Otolaryngology‐led OBT protocol at a public safety net hospital, with low complication rates while saving on staffing and equipment costs.^7^ Their OBT surgical team included an Otolaryngology surgical resident as the first assist.^7^ In our study, OBT could be performed with only 1 otolaryngology surgeon and a nonphysician staff (OR technician) as first assist, with an ICU circulating nurse and RT composing the rest of our OBT team. We were successfully able to train nursing staff and ICU RT in a hospital with no prior OBT program.

OBT may be up to 10 times less costly than ORT.^13^ Potential savings of performing tracheostomies at the ICU bedside compared to in the OR were found in 1 study to be $4575 per case.^6^ Costs related to practitioner fees and equipment costs can be $1024 lower for OBT than for ORT in similar staff‐limited settings.^7^ A study at a tertiary hospital demonstrated OBT freed 279 hours of OR occupancy in 1 year.^12^ In a smaller community hospital, this may have an even more significant impact. Smaller community and rural hospitals have lower operative margins of profit than tertiary medical centers.^21^ OR availability at smaller community hospitals may be limited, and the opportunity cost of another operation that could have utilized the OR might be another important consideration. By leaving the availability of an OR, OBT allows for scheduling more procedures that can be important sources of revenue for a not‐for‐profit community hospital, in addition to preserving OR staff and resources. On one hand, OBT takes OR staff away from the OR to transport equipment to the ICU; on the other hand, the transportation of an ICU patient on a vent to and from the OR requires ICU and RT to leave transportation and represents a potential greater resource utilization and safety risk to the patient.^22^ Future studies should investigate these unmeasured costs and risks associated with tracheostomy beyond the operation itself.

### Limitations

This study has several limitations. The community hospital affiliate is located in an urban setting, and rural community hospitals may experience different barriers to program implementation. Extended long‐term follow‐up information was limited given the recency of the study period, and many patients were lost to follow‐up. The preliminary statistical analysis of this study is likely underpowered given the small pilot sample, though exact methods may help to mitigate this.^20^ The ORT group is disproportionately smaller due to increased provider preference for OBT since the implementation of the OBT program in 2016. However, the rates of the safety of ORT are well‐established by multiple studies,^23‐26^ and the primary purpose of this study was not to evaluate the safety or outcomes of ORT but rather to demonstrate OBT in this community setting are comparable. The performing surgeon was not controlled for within or across tracheostomy location groups, and the data may not account for variations in certain practice patterns across different providers. Operating costs to the hospital were crudely estimated by key hospital financial personnel based on operating expense rates for the 2021 fiscal year; the exact itemization of expenses that composed this calculation could not be obtained from hospital financial leadership. Additionally, this cost does not include staff utilization. However, we believe this crude cost estimation may still be useful as an initial metric for the lone community Otolaryngologist interested in opening a discussion about OBT with staff collaborators and hospital administrators. Despite these limitations, this pilot study suggests an OBT program can be successfully implemented by a single Otolaryngologist in a community hospital setting with low complication rates and possible cost savings to the hospital.

## Conclusion

An OBT program can be implemented by a single surgeon at a community hospital to optimize hospital resources and operate cost‐efficiency while maintaining patient safety. We present a model of an Otolaryngologist to create an OBT program at a community hospital with limited staff and resources.

## Author Contributions

**Ryan S. Ziltzer**, study conception and design, acquisition of data, analysis and interpretation of data, drafting of the manuscript, critical revision; **Noah M. Millman**, acquisition of data, analysis and interpretation of data, drafting of the manuscript, critical revision; **Jorge Serrano**, acquisition of data, drafting of the manuscript; **Mark Swanson**, acquisition of data, analysis and interpretation of data, critical revision; **Karla O'Dell**, study conception and design, acquisition of data, analysis and interpretation of data, drafting of the manuscript, critical revision.

## Disclosures

### Competing interests

None.

### Sponsorships

None.

### Funding source

None.
